# Local and neighboring patch conditions alter sex‐specific movement in banana weevils

**DOI:** 10.1002/ece3.1818

**Published:** 2015-11-20

**Authors:** Dominique Carval, Benjamin Perrin, Pierre‐François Duyck, Philippe Tixier

**Affiliations:** ^1^ CIRADUPR GECOF‐97285Le LamentinMartiniqueFrance; ^2^ CIRADUMR PVBMTF‐97410Saint‐PierreLa RéunionFrance; ^3^ Departamento de Agricultura y AgroforesteriaCATIECR‐30501TurrialbaCosta Rica

**Keywords:** Behavioral heterogeneity, *Cosmopolites sordidus*, density‐dependent movement, intrasexual competition, sex ratio, sex‐biased movement

## Abstract

Understanding the mechanisms underlying the movements and spread of a species over time and space is a major concern of ecology. Here, we assessed the effects of an individual's sex and the density and sex ratio of conspecifics in the local and neighboring environment on the movement probability of the banana weevil, *Cosmopolites sordidus*. In a “two patches” experiment, we used radiofrequency identification tags to study the *C. sordidus* movement response to patch conditions. We showed that local and neighboring densities of conspecifics affect the movement rates of individuals but that the density‐dependent effect can be either positive or negative depending on the relative densities of conspecifics in local and neighboring patches. We demonstrated that sex ratio also influences the movement of *C. sordidus*, that is, the weevil exhibits nonfixed sex‐biased movement strategies. Sex‐biased movement may be the consequence of intrasexual competition for resources (i.e., oviposition sites) in females and for mates in males. We also detected a high individual variability in the propensity to move. Finally, we discuss the role of demographic stochasticity, sex‐biased movement, and individual heterogeneity in movement on the colonization process.

## Introduction

Movement, defined as a change in the spatial location of an individual (Schellhorn et al. 2014), is necessary for the survival, reproduction, and dispersal of many organisms. Understanding the mechanisms underlying the movements and the spread of a species across time and space is a major concern of ecology. In particular, the stochasticity in population dynamics occurring at the colonization front may generate variability in local densities and sex ratios (Miller and Inouye 2013). Moreover, the individual variability in movement response is likely to enhance the effect of demographic stochasticity. Variation in movement may be the consequences of complex interactions between the behavior of individuals and environmental cues (Matthysen 2012). Theory predicts that, in large populations in spatially and temporally variable environments, movement leads to an ideal free distribution of individuals (Holt and Barfield 2001). In such situations, density‐dependent movement may result in an even fitness expectancy of conspecifics across space (Holt and Barfield 2001; Clobert et al. 2009). The density‐dependent effect can be either positive or negative depending on the strength of the autocorrelation of the environmental stochasticity. When this environmental stochasticity is strong, the density is perceived by individuals as a proxy for conspecific competition, and positive density‐dependent movement is expected (Baguette et al. 2011). Conversely, when the autocorrelation is weak, the density is perceived by individuals as a proxy for patch quality, and negative density‐dependent movement is expected (Baguette et al. 2011). Positive density‐dependent movement has been well documented by experimental studies (for a review, see Bowler and Benton 2005). Although negative density‐dependent movement is also commonly found for mammals and birds (for a review, see Matthysen 2005), it has seldom been documented for insects (Roland et al. 2000; Chaput‐Bardy et al. 2010; Baguette et al. 2011). Experimental studies on how density‐dependent movement is affected by the biotic and abiotic environment in which individuals evolve have not yet established a comprehensive framework linking density dependence and spatial population dynamics. In the current study, we explore how density‐dependent movement is affected by the conditions in local and neighboring patches.

In addition to being influenced by population density, the movement decision may also be influenced by inbreeding, kin competition, intra‐ and interspecific competition, or intra‐ and intersexual competition (Gandon 1999; Bowler and Benton 2005; Clobert et al. 2009; Trochet et al. 2013). Sex‐biased movement, the difference in movement between males and females, is recognized as being a consequence of any divergent evolutionary responses between sexes (Trochet et al. 2013). Sex‐biased movement may thus result from male–male competition for mating, female–female competition for habitat quality (e.g., competition for oviposition sites; Albrectsen and Nachman 2001), or male–female interactions (e.g., female harassment by males; Le Galliard et al. 2005). In a theoretical model, Perrin and Mazalov (2000) predicted that the movement rates are higher in the sex that suffers more from local competition, which is generally the less abundant sex. According to this model, the effect of sex ratio on the movement rate of an individual should depend on the sex of the individual. Because the local density affects local competition and patch attractiveness and because sex ratio influences intra‐ and intersexual competition, variations in densities and sex ratios and interactions between these factors are thus expected to condition the sex‐biased movement.

Many theoretical studies on movement have focused on the mechanisms explaining differences in movement rates between sexes (Meier et al. 2011). Sex ratio and density are not static, and their variations could lead to spatial variation in other biological variables. A model of the evolution of movement showed that sex ratio and population density influence the triggering of movement (Hirota 2007). A spatial model showed that the feedback between spatial variations in sex ratio and densities could lead to similar movement behaviors between sexes (Meier et al. 2011).

However, theoretical studies that focus on the evolution of sex‐biased movement have generally assumed that movement is a fixed trait in individuals, which is not always the case (Bowler and Benton 2005). In addition, most experimental studies have examined the effect of sex‐biased movement on the variation in the local sex ratio of populations and have not considered how sex‐biased movement is a flexible response to variation in the local sex ratio and, hence, to variation in intra‐ and intersexual competition. To our knowledge, only two studies have assessed the movement of adults in response to manipulated sex ratio under seminatural conditions (Le Galliard et al. 2005; Trochet et al. 2013), and the authors of these studies concluded that the adult sex ratio is regulated by a balance between intra‐ and intersexual competition rather than by mate availability as suggested by theory (Perrin and Mazalov 2000). To close the gap between theoretical prediction and experimental observations, the current research assessed the influence of intra‐ and intersexual competition on the movement response of individuals. This was done by varying the sex ratio of the studied organism, the banana weevil, *Cosmopolites sordidus* (Germar).

The current paper aims to understand the factors that influence the movement decisions and therefore the colonization potential of the banana weevil *C. sordidus* (Germar). In an experiment that used populations of *C. sordidus* and focused on the departure stage of movement, we tested the theoretical prediction that sex‐biased movement strategies tend to balance intra‐ and intersexual competition. We studied the effects of individual phenotype (sex) and local and neighboring patch conditions (variations in conspecific densities and sex ratios) on the movement probability of *C. sordidus* in a “two patches” environment using RFID methods. This experiment determined (1) how the local and neighboring densities of conspecifics affect the movement rates of individuals; (2) how the local and neighboring sex ratios affect the movement rates of individuals; (3) whether banana weevils exhibit sex‐biased movement to balance intra‐ and intersexual competition; and (4) the individual variability in movement behavior.

## Materials and Methods

### Biological model: *Cosmopolites sordidus*

*Cosmopolites sordidus* is the most important insect pest of bananas and plantains. This weevil originated in the Indo‐Malaysian region (Gold et al. 2001), which coincides with the area of origin of banana (Stover and Simmonds 1987). *Cosmopolites sordidus* is a walking insect that is sedentary during daylight hours and is active but cryptic at night (Cuillé 1950; Gold et al. 2001). The adult length ranges from 8.8 to 15.0 mm, and females are about 20% longer than males (Gold et al. 2001). The adult is the only stage of *C. sordidus* that moves. Although adults have functional wings, reports of flight are scarce (Gold et al. 2001). *Cosmopolites sordidus* displays limited long‐distance movement (Gold et al. 2001), but short‐distance movement between banana plants may be important; the mean and maximum movement distances per night were reported to be 0.5 and 9 m, respectively (Vinatier et al. 2010). Adults can live up to 2 years (Gold et al. 2001). The mean number of laid eggs per female per week is 2.4 without intraspecific competition and 0.8 with intraspecific competition (Treverrow et al. 1992). Males guard females after mating to prevent further mating (Gold et al. 2001). Females may oviposit for up to 11 months without mating again (Cuillé 1950) and may lay up to 100 eggs following a single mating (Treverrow et al. 1992). The sex ratio at birth is close to 1:1 but ranges from 1.7:1 to 1:2.2 (Gold et al. 2001). Population densities in banana crops are highly variable, are greatly affected by management strategies, and may exceed 300 individuals per mat (Gold et al. 2001).

### *Cosmopolites sordidus* capture, sexing, and marking

We collected 860 *C. sordidus* adults from one banana field by using banana pseudostem traps supplemented with the sordidine pheromone *Cosmolure* ^*®*^ (Budenberg et al. 1993). The banana field was located on the Campus Agro‐Environnemental Caraïbe (CAEC), Petit Morne, Martinique, West Indies, and the collecting was approved by the Head of the CAEC, Christian Chabrier. The pseudostem traps were laid on the ground near banana plants to attract weevils. All collected individuals were sexed based on rostrum punctuation and curvature of the last abdominal sternite (Gold et al. 2001). Before the experiment began, weevils were maintained in translucent plastic boxes (80 × 40 × 40 cm) with pseudostem pieces at 25°C and with a relative humidity of 85% for a maximum of 15 days. Twenty‐four hours before being released into the experimental patches (see next section), males and females were marked with an RFID tag (ref: TXP148511B; Biomark Inc., Boise, ID). The tag was attached to one end of an 8.0‐mm long piece of Dyneema^®^ fishing line (ref: Sufix 22.7 kg, 0.34 mm diameter) and the other end of the line was attached to the individual with cyanoacrylate glue (Super Glue^®^, Ontario, Canada); a previous study showed that this method of tagging did not affect weevil mobility (Vinatier et al. 2010). The tagged individuals were maintained together in a translucent plastic box with pieces of pseudostem and a 12‐h/12‐h photoperiod until they were used in the experiment.

### Experimental setup

The experimental system consisted of two plastic patches (each patch was 50 cm in diameter) that were linked by a corridor (a transparent plastic tube) that was 1 m long, 7 cm wide, and 4 cm high. The patches were filled with washed sand, and the corridor was located to enable movement of weevils between the sand surface and the corridor. One plantain bulb (*Musa*, AAB, French Horn, collected in the field at Petit Morne experimental farm, Martinique, West Indies) was placed in the center of each patch and was changed after 10 days. Movements of individuals were recorded by antennas located on the corridor and 10 cm distant from each patch; the antennas were connected to RFID readers (FS2001F‐ISO reader, Biomark Inc.; see the Supporting information, Fig. S1). Tag detection was restricted to a mean distance of 3.74 cm (±SE 0.86) from the antenna by using each antenna at 10% of its capacity. The experimental system was maintained in a climate‐controlled room at 25°C and with a relative humidity of 85% and a 12‐h/12‐h photoperiod (the photoperiod matched that of the natural environment).

### Experiment

We defined the local patch as the patch where an individual is present at time *t*. Similarly, we defined the neighboring patch as the patch where the individual is absent at time *t*. We defined a movement as the crossing from one patch to another by an individual. A large number of combinations of local and neighboring densities (i.e., treatments) were created by varying the initial number of individuals from 4 to 50 in the patch of release. The initial sex ratio was 1:1 for each treatment (hereafter, we called the “sex ratio” as the number of males divided by the total number of individuals). To minimize the effect of individual variability in behavior, we used each individual only one time, and we replicated each treatment 10 times (when initial weevil numbers were 4, 6, or 10) or four times (when initial weevil numbers were 16, 20, 24, 30, 40, or 50). We alternated the patch where *C. sordidus* individuals were placed between replicates. Each replicate was started 5 min before the beginning of the artificial night by placing the desired number of individuals randomly around the plantain bulb in the release patch. The same individual could not be detected by the same antenna during the following 60 sec. The recorder registered the individual identification tag number and time of detection.

### Real‐time dynamics

Local density *x*_*i*_(*t*) is the number of individuals in patch *i* at time *t*. To determine *x*_*i*_(*t*), we performed an algorithm that divided the data according to the time of night. For each replicate, we retrieved the position of each marked weevil, and consequently we knew the exact local density *x*_*i*_(*t*) and the local sex ratio of patch *i* at any time *t*. From the population dynamics of the patches in the original data, we calculated the local density *x*_*i*_(*t*) of each patch as the mean of all the local densities observed in the time interval [*t*;* t + *Δ*t*] in patch *i*. We applied the same procedure for calculation of the sex ratio. We assigned the value 1 when the weevil had moved between patches during Δ*t* and the value 0 when the weevil had not moved between patches. We thus assumed that the decisions of individuals to move were related to *x*_*i*_(*t*). The algorithm was performed for Δ*t* values of 5, 10, 15, 20, 25, 30, and 60 min to assess the robustness of the method and consistency between results.

### Statistical analysis

We used generalized linear mixed models (GLMMs) to analyze the effects of patch conditions and sex on the probability of movement. Binomial GLMMs describe the logit of the response variable *p* as a function of the linear combination of an intercept, fixed effects ($x_{\beta }^{\prime }$), and random effects ($z_{b}^{\prime }$), which are assumed to follow a normal distribution centered at 0 and with variance *σ* ^2^. A global GLMM took the general form of:

$$\text{logit}\left(p\right)=\alpha +x_{\beta }^{\prime }+z_{b}^{\prime }\;b∼N\left(0,\sigma^{2}\right).$$

Probability of movement was analyzed using GLMMs with binomial error (logit link function; Jaeger 2008) as a function of the following fixed effects: time, density of the local patch, density of the neighboring patch, sex of individuals, sex ratio of the local patch, sex ratio of the neighboring patch, bulb age, and interactions. Two crossed, random intercept effects were included: the individuals and the replicates. The individual‐level random intercept captures potential differences in the base probability that weevils move (Jaeger 2008). The replicate‐level random intercept captures the potential variance between replicates.

To select models, we first tested for collinearity (i.e., correlation) between covariates using the variance inflation factor (VIF) method (Zuur et al. 2010). We then examined the need to include the random effects using likelihood ratio tests (LRTs; Bolker et al. 2009; see the Supporting information, Table S2) and estimated the dispersion parameter as being the square root of the penalized residual sum of squares divided by the number of observations. After identifying the appropriate random effect structure for the global GLMM, we removed nonsignificant fixed effect parameters in a backward‐stepwise process using LRTs. We first removed nonsignificant interactions and then removed nonsignificant variables. The selection procedure was continued until a model was found in which all effects were significant (Zuur et al. 2009).

All GLMMs were estimated using the glmer function in the “lme4” package (Bates et al. 2012), in which the maximum likelihood of parameters is approximated by the Laplace method (Bolker et al. 2009).We also performed a Bayesian estimation of parameters of the best GLMM using the MCMCglmm package (Hadfield 2010); see the Supporting information, Tables S1 and S3) based on the deviance information criteria. MCMC offers the advantage of providing confidence intervals on parameters in a way that averages the uncertainty in the fixed and random effect parameters (Bolker et al. 2009). Statistical analyses were performed with R 2.15.0 (R Development Core Team 2012), and an alpha level of 0.05 was used.

## Results

### Night‐time activity

**Figure 1:**
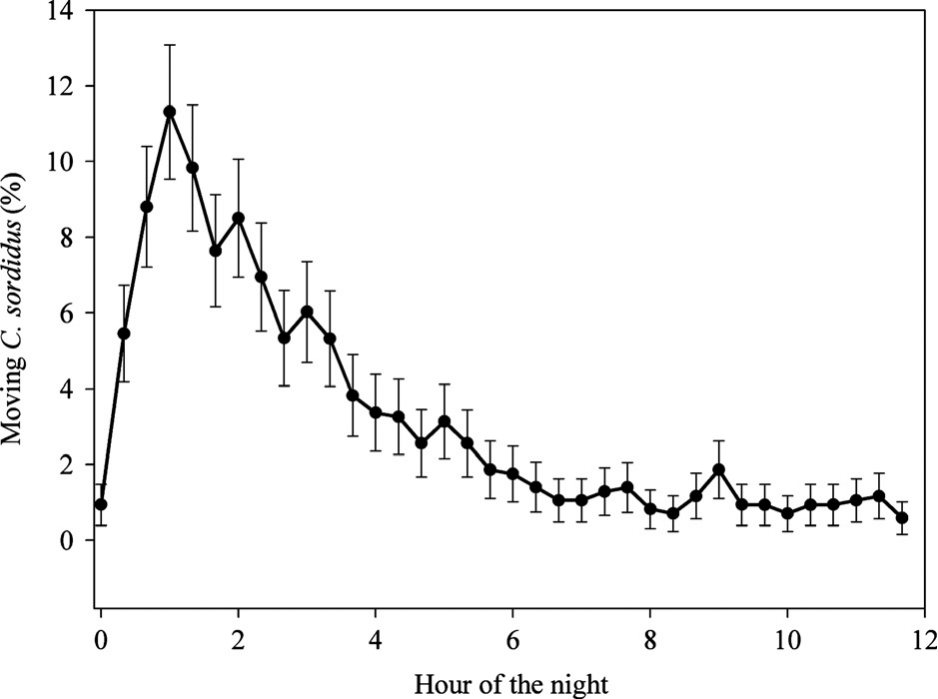
Percentage of *Cosmopolites sordidus* that moved as a function of time of night. Values are means of replicates with a range of initial densities in local and neighboring patches, and error bars represent the 95% confidence intervals.

For all densities of weevils, the movement of individuals during the night peaked after about 1 h and then decreased; few weevils moved during the last 2 h of the 12‐h night (Fig. 1). The mean number of crossings per individual was 1.24 per night for females and 1.1 per night for males. The mean (±SE) duration of crossings was 5.29 ± 2.45 min, *N *=* *882.

### Movement behavior

**Figure 2:**
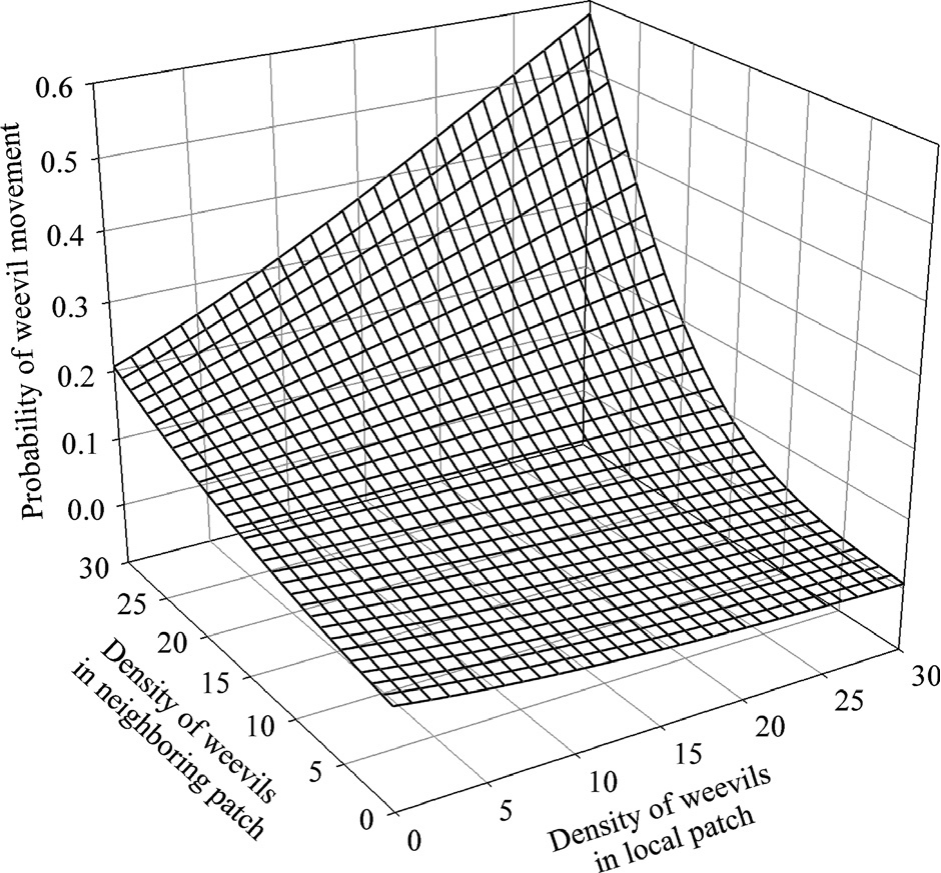
Influence of the local and neighboring densities on the probability of *Cosmopolites sordidus* movement. The time was set at 120 min after the start of darkness, and the initial sex ratio (number of males/number of individuals) was 0.5 in both the local and neighboring patches.

**Figure 3:**
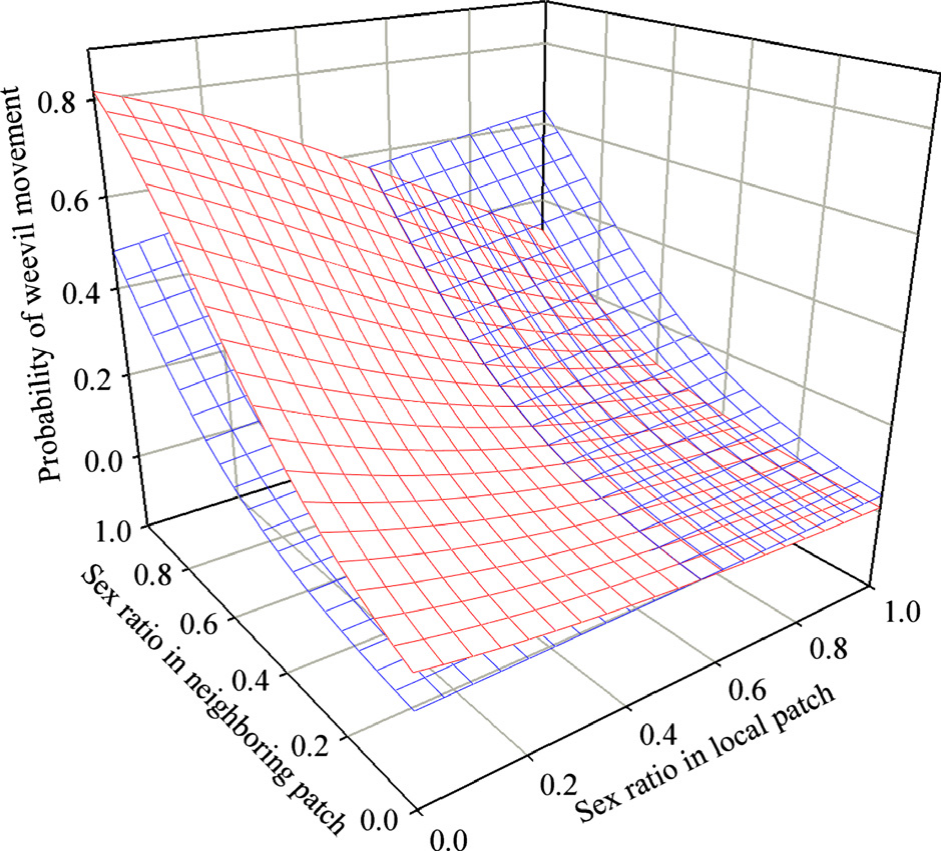
Influence of the local and neighboring patch sex ratios (number of males/number of individuals) on the probability of *Cosmopolites sordidus* movement. Red area: movement response of females. Blue area: movement response of males. The time was set at 120 min after the start of darkness, and the local and neighboring densities were set at 20 individuals.

According to GLMMs, the probability that an individual moved was significantly affected by the hour of the night (LRT: *P* < 0.001, *χ* ^2^ **=* *198, df* *=* *1), local density (LRT: *P *<* *0.001, *χ* ^2^ **=* *26.828, df* *=* *1), neighboring density (LRT: *P *<* *0.001, *χ* ^2^ **=* *55.818, df* *=* *1), local sex ratio (LRT: *P *=* *0.0012, *χ* ^2^ **=* *10.502, df* *=* *1), and neighboring sex ratio (LRT: *P *<* *0.001, *χ* ^2^ **=* *125.05, df* *=* *1). Quadratic terms of the hour of the night significantly affected the probability of movement (LRT: *P *<* *0.001, *χ* ^2^ **=* *33.588, df* *=* *1). The probability of movement was also significantly affected by interactions between local and neighboring densities (LRT: *P *<* *0.001, *χ* ^2^ **=* *31.065, df* *=* *1), between local density and local sex ratio (LRT: *P *<* *0.001, *χ* ^2^ **=* *24.494, df* *=* *1), between neighboring density and neighboring sex ratio (LRT: *P *=* *=* *0.0046, *χ* ^2^ **=* *8.042, df* *=* *1), and between neighboring density and local sex ratio (LRT: *P *<* *0.001, *χ* ^2^ **=* *24.759, df* *=* *1). The sex of an individual did not significantly affect its probability of movement (LRT: *P *=* *0.245, *χ* ^2^ **=* *1.352, df* *=* *1), but was retained in the final model because of the significant interaction between sex and local sex ratio (LRT: *P *<* *0.001, *χ* ^2^ **=* *46.754, df* *=* *1). The age of bulbs did not significantly affect the probability of movement (LRT: *P *=* *0.390, *χ* ^2^ **=* *0.738, df* *=* *1) and was removed from the final model. The probability of movement was not significantly affected by the interactions between local density and neighboring sex ratio (LRT: *P *=* *0.580, *χ* ^2^ **=* *0.306, df* *=* *1) or between sex and neighboring sex ratio (LRT: *P *=* *0.177, *χ* ^2^ **=* *1.8206, df* *=* *1), and these terms were removed from the final model. Banana weevils responded linearly to the density of individuals both in the local and the neighboring patch, in that the probability of movement from the local patch decreased with local density and increased with neighboring density (Fig. 2, Table 1). When the density was high in both patches, the probability of movement was high (Fig. 2). The probability that females moved increased as female abundance (relative to that of males) increased, and the probability that males moved increased as male abundance (relative to that of females) increased (Fig. 3). In other words, the probability of female movement increased when the local sex ratio was female biased, whereas the probability of male movement increased when the local sex ratio was male biased. Both males and females showed a higher movement probability when the neighboring patch was male biased. The interaction between sex ratios and the local and neighboring densities modified the strength of attractiveness of the patches but not the general trends (see the Supporting information, Figs. S2–S4). The variance in the individual propensity to move was 0.801 (Table 1). Estimates of predictors and random terms were consistent between the frequentist and the Bayesian methods (see the Supporting information, Table S1). With the exception of a Δ*t* value of 5 min, results were consistent across the range of Δ*t* values used to produce the data describing real‐time dynamics, and the best GLMMs were qualitatively similar although there were some discrepancies in the absolute values of the estimated parameters (see the Supporting information, Tables S4 and S5).

**Table 1:** MCMC *β* estimates for explanatory variables with significant effects on the probability of movement and for random variables after backward selection (likelihood ratio tests)

	Estimate	95% Confidence interval	
		Lower limit	Upper limit
Fixed effects			
Intercept	−3.401332	−4.705853	−3.188688
Time	−0.011571	−0.015065	−0.011483
Local density	−0.025927	−0.050410	−0.001394
Neighboring density	0.061182	0.027789	0.114491
Sex (male)	−1.509120	−2.253434	−1.229263
Local sex ratio	1.545665	0.695947	2.751488
Neighboring sex ratio	2.377253	1.996919	3.312317
Time2	0.000008	0.000006	0.000011
Local density × Neighboring density	0.004087	0.002921	0.006119
Local density × Local sex ratio	−0.075356	−0.133258	−0.056838
Neighboring density × Local sex ratio	−0.094375	−0.161437	−0.067494
Neighboring density × Neighboring sex ratio	0.063923	0.026191	0.120073
Sex (Male) × Local sex ratio	2.775510	2.249468	4.037539
Random effects			
Individual	0.801059	0.74401	1.242563
Replicate	0.216753	0.11186	0.570949

For each explanatory variable, a positive (negative) effect means an increase (decrease) in the probability of *Cosmopolites sordidus* movement.

## Discussion

The current study indicates that movement of *C. sordidus* from the local patch decreases with local conspecific density and increases with neighboring conspecific density. These results support the hypothesis that individuals use the local conspecific density as a proxy for patch quality and suggest that individuals somehow obtain information on the neighboring patch quality through conspecific density. The information obtained from the neighboring patch is probably provided by a pheromone. *Cosmopolites sordidus* males produce a sordidine aggregation pheromone, which is likely used by individuals to assess conspecific density in surrounding patches (Budenberg et al. 1993). In an experiment involving habitat‐quality manipulation, Baguette et al. (2011) documented negative density‐dependent movement in a metapopulation of the butterfly *Boloria eunomia*. In that experiment, movement from high‐quality patches decreased in spite of an increase of immigrants from other patches, which also suggests that individuals may gather information on the quality of surrounding patches. Interestingly, we found that as local and neighboring densities increased, the movement response of local individuals to local density reversed. Indeed, as densities increased in both local and neighboring patches, individuals in local patches displayed a positive density‐dependent movement. These results suggest that individuals may also perceive local conspecific densities as a proxy of competiveness and may perceive neighboring conspecific densities as a proxy for patch quality. Therefore, our results show that density‐dependent movement in *C. sordidus* is context dependent, which agrees with current dispersal theory (Bowler and Benton 2005; Clobert et al. 2009). Moreover, the switch in the nature of the density‐dependent effect (from negative to positive) exhibited by *C. sordidus* supports the idea that movement is fitness dependent (Ruxton and Rohani 1998). Ruxton and Rohani (1998) showed that the decision to move in a metapopulation depends not only on local density but also on the density of patches where the dispersing individuals could settle. Moreover, because the food resource (the bulb) was the same in each patch of the current study, our results suggest that local and neighboring conspecific densities might greatly affect movement decisions in a homogeneous environment, like that provided by intensive banana crops. The switch from negative density‐dependent movement toward positive density‐dependent movement also suggests that colonization may lead to a clustered population where individuals tend to remain in the same patches until neighboring patches have higher densities. He and Alfaro (1997) found that pine weevils are aggregated in the early phases of colonization, are then randomly dispersed, and finally are regularly distributed in space as colonization proceeds. In summary, the decision to move and, subsequently, the decision to settle depend on both the local and neighboring patch conditions.

We found that a biased sex ratio in the local patch results in the movement of the more abundant sex. This finding is consistent with the theoretical prediction that movement patterns depend on sex ratio (Perrin and Mazalov 2000) and that intrasexual competition may buffer variations in sex frequency and lead to a balanced sex ratio in the population. The female‐biased movement response, which we observed when the local sex ratio was female biased, is consistent with the hypothesis that females experience local competition, for example, for oviposition sites (Perrin and Mazalov 2000). *Cosmopolites sordidus* females lay more eggs when female densities are low rather than high (Gold et al. 2002). Moreover, the decision to oviposit is likely to depend on the presence of conspecific females rather than on the detection of eggs at the oviposition site (Gold et al. 2002). This result supports the assumption that the decision to oviposit by females depends on the real‐time local number of females rather than on the numbers of eggs and larvae on the banana corm. This seems reasonable because as the number of females increases, competition among the progeny will increase and progeny fitness will decrease, which is consistent with the faster development of *C. sordidus* larvae at higher egg densities (Gold et al. 2002). Otherwise, the male‐biased movement response, which we observed when the local patch was male biased, is consistent with competition among males for female mates (Greenwood 1980). In support of this inference, males exhibit guarding behavior to prevent further mating of females by competing males (Gold et al. 2001). To our knowledge, only two experimental studies, one with the lizard *Lacerta vivipara* (Le Galliard et al. 2005) and the other with the butterfly *Pieris brassicae* (Trochet et al. 2013), have focused on the effect of variation in sex ratio on adult movement. In these previous studies, the animals exhibited no tendency to balance population sex ratios. In the study with the lizard *L. vivipara*, movement of females tended to be higher when the local sex ratio was female biased, which is consistent with the local resource competition hypothesis (Perrin and Mazalov 2000); male lizards, however, did not display a higher movement rate when the local sex ratio was male biased, suggesting that local mate competition did not favor male movement as predicted by theory (Greenwood 1980). In the study with the butterfly *P. brassicae*, movement of males increased with the ratio of males/females in the patch of departure, which agrees with local mate competition, but females did not show an increased propensity to leave the patch when local sex ratio was female biased, which disagrees with local resource competition. The differences in movement patterns in *C. sordidus* versus *L. vivipara* and *P. brassicae* might be explained by seasonal constraints. Although *C. sordidus* has evolved in a tropical climate without seasonal constraints on reproduction and movement, *L. vivipara* and *P. brassicae* have evolved in temperate climates and are constrained by seasonality; seasonality generates different selective pressures on life history traits, including movement.

We have shown here that conspecific density greatly affects the movement decision of *C. sordidus*. We have also shown that variation in local sex ratio modifies sex‐biased movement, indicating that sex‐biased movement is not fixed in *C. sordidus*. The bias in *C. sordidus* movement (i.e., movement is greater for the more abundant sex) may explain the balanced sex ratio generally observed in large natural populations of banana weevils that reside in the same field (Gold et al. 2001). We also found that *C. sordidus* individuals moved more when the local density was low, which could reduce the rate of spread toward new, empty habitats. Demographic stochasticity is then likely to act on *C. sordidus* colonization of banana crops. Cadet et al. (2003) stressed the importance of considering demographic stochasticity in the evolution of movement. They showed that demographic stochasticity may provide enough variability in the density of individuals in patches to favor the evolution of movement, even if the environment itself is homogeneous in time and space. Their results suggest that selection for movement depends essentially on the crowding effect in high‐density patches. Here, we found that movement is sex‐biased only when the sex ratios were unbalanced. Demographic stochasticity is likely to produce a large variation in sex ratio in the first section of a field invaded by *C. sordidus* and, hence, to cause sex‐biased movement, which may affect the rate of colonization. Miller and Inouye (2013) cautioned against using the noisiness of demographic stochasticity without considering the effects of sex‐biased movement. Through a combination of experimental and modeling approaches, they showed that sex‐biased movement may alter invasion velocity but that the effect is diluted by demographic stochasticity when the sex bias in movement is low.

Variability in local densities and sex ratios is not the only source of stochasticity in the movement process. The individual variation in the propensity to move might be important, especially when it is combined with demographic stochasticity at the edge of the colonization front. In our experiments, we detected substantial variability among individuals in the propensity to move (the variance of the individual‐level random effect), but because individuals were tested only once in the experiment, we cannot make inferences about the possible existence of disperser and nondisperser phenotypes in this species. Individual‐based modeling (IBM) is well suited to simulate the dynamics of the spatial spread of a species (South and Kenward 2001; Vinatier et al. 2009). Incorporating variance in the individual propensity to move in an IBM would enable researchers to test the relative effect of demographic stochasticity and individual variance on the rate of colonization. Behavioral heterogeneity in dispersers is linked to dispersal syndromes, that is, to the covariation of multiple traits associated with dispersal (which is a particular type of movement; see Schellhorn et al. 2014; for details on terminology) (Clobert et al. 2009; Matthysen 2012). Ducatez et al. (2012) found variation in movement among individuals of the butterfly *P. brassicae*, suggesting the existence of a mobility syndrome in this species. Extending the experimental methods described in the current study by repeatedly measureing movements of the same individuals would generate data that could be used to determine whether *C. sordidus* has movement syndromes resulting in disperser and nondisperser phenotypes.

In summary, using *C. sordidus* as a model, we have shown that local and neighboring densities of conspecifics can affect the movement rates of individuals but that the sign of the density‐dependent movement may be context dependent, that is, may depend on the relative densities of conspecifics in local and neighboring patches. We demonstrated that variation in sex ratios also influences the movement of *C. sordidus*, and we inferred that sex‐biased movement would tend to balance the sex ratio and to minimize intrasexual competition. Finally, we detected a high variance in the propensity of individuals to move, and we suspect the possible existence of disperser and nondisperser phenotypes in *C. sordidus*. Otherwise, the maximal number of crossings observed in our experiment is consistent with the maximal distance of 9 m/day in the field reported by Vinatier et al. (2010), which supports the relevance of our experimental setup for the study of *C. sordidus* movement. A recent study of *C. sordidus* in a heterogeneous environment showed the existence of a habitat‐dependent kernel (Vinatier et al. 2011). Extending the experimental system used in the current study to a metapopulation experiment that combines variation in patch environment, weevil density, and weevil sex ratio would be useful for increasing our understanding of the colonization process in *C. sordidus*.

## Conflict of Interest

None declared.

## Data Deposit and Material Sharing

The original dataset and the data describing real‐time dynamics for each value of *Δt* can be downloaded at: http://knb.ecoinformatics.org/knb/metacat?action=read&qformat=knb&docid=knb.341.1 and http://knb.ecoinformatics.org/knb/metacat?action=read&qformat=knb&docid=knb.345.1

## Supporting information


**Table S1.** Laplacian and MCMC *β* estimates for explanatory variables with significant effect on the probability to move and for random variables after backward selection (likelihood ratio tests).
**Table S2.** Likelihood ratio tests for the significance of adding “individual” and “replicate” random effects to the global model (in bold).
**Table S3.** Model selection based on the deviance information criterion (DIC) (MCMCglmm R package; Hadfield 2010).
**Table S4.** Estimates of the best GLMMs (backward selection using likelihood ratio tests) across the range of Δ*t* values from 5 to 20 min.
**Table S5.** Estimates of the best GLMMs (backward selection using likelihood ratio tests) across the range of Δ*t* values from 25 to 60 min.

**Figure S1.** Experimental design for the study of the behavior of *Cosmopolites sordidus*, the banana weevil.
**Figure S2.** Influence of the local densities and local sex ratio (number of males/number of individuals) on the probability of *Cosmopolites sordidus* movement.
**Figure S3.** Influence of the neighboring densities and local sex ratio (number of males/number of individuals) on the probability of *Cosmopolites sordidus* movement.
**Figure S4.** Influence of the neighboring densities and neighboring sex ratio (number of males/number of individuals) on the probability of *Cosmopolites sordidus* movement.
