# Supplementary material for: Local and neighboring patch conditions alter sex‐specific movement in banana weevils
Source: Ecol Evol. 2015 Nov 20;5(23):5735–43. doi: 10.1002/ece3.1818 (PMC4813121; doi:10.1002/ece3.1818)
Supplement: Supplementary file 1 — Table S1. Laplacian and MCMC β estimates for explanatory variables with significant effect on the probability to move and for random variables after backward selection (likelihood ratio tests). Table S2. Likelihood ratio tests for the significance of adding “individual” and “replicate” random effects to the global model (in bold). Table S3. Model selection based on the deviance information criterion (DIC) (MCMCglmm R package; Hadfield 2010). Table S4. Estimates of the best GLMMs (backward selection using likelihood ratio tests) across the range of Δt values from 5 to 20 min. Table S5. Estimates of the best GLMMs (backward selection using likelihood ratio tests) across the range of Δt values from 25 to 60 min. [file ECE3-5-5735-s001.docx]

**Table S1.** Laplacian and MCMC β estimates for explanatory variables with significant effect on the probability to move and for random variables after backward selection (likelihood ratio tests). For each explanatory variable, a positive (negative) effect means an increase (decrease) in the probability of movement.

|  | **GLMM** | | **MCMC GLMM** | | |
| --- | --- | --- | --- | --- | --- |
|  | Estimate | Standard error | Estimate | Confidence interval | |
|  |  |  |  | Lower limit | Upper limit |
| **Fixed effects** | | | | | |
| Intercept | -3.493400 | 0.354700 | -3.401332 | -4.705853 | -3.188688 |
| Times | -0.012366 | 0.000857 | -0.011571 | -0.015065 | -0.011483 |
| Local density | -0.024412 | 0.011316 | -0.025927 | -0.050410 | -0.001394 |
| Neighboring density | 0.071716 | 0.020016 | 0.061182 | 0.027789 | 0.114491 |
| Sex (Male) | -1.565800 | 0.243700 | -1.509120 | -2.253434 | -1.229263 |
| Local sex ratio | 1.639700 | 0.484500 | 1.545665 | 0.695947 | 2.751488 |
| Neighboring sex ratio | 2.507400 | 0.311000 | 2.377253 | 1.996919 | 3.312317 |
| Times² | 0.0000087 | 0.0000012 | 0.000008 | 0.000006 | 0.000011 |
| Local density * Neighboring density | 0.004166 | 0.000770 | 0.004087 | 0.002921 | 0.006119 |
| Local density * Local sex ratio | -0.089406 | 0.018255 | -0.075356 | -0.133258 | -0.056838 |
| Neighboring density * Local sex ratio | -0.105005 | 0.021101 | -0.094375 | -0.161437 | -0.067494 |
| Neighboring density * Neighboring sex ratio | 0.061391 | 0.021809 | 0.063923 | 0.026191 | 0.120073 |
| Sex (Male) * Local sex ratio | 2.867800 | 0.429600 | 2.775510 | 2.249468 | 4.037539 |
| **Random effects** | | | | | |
| Individual | 0.78505 | - | 0.801059044 | 0.74401 | 1.242563015 |
| Replicates | 0.21294 | - | 0.216753271 | 0.11186 | 0.570949829 |

**Table S2.** Likelihood ratio tests for the significance of adding ‘individual’ and ‘replicate’ random effects to the global model (in bold). A *p*-value < 0.05 indicates rejection of the simpler model in favor of the more complicated model (i.e., that with more random effects). Dispersion parameter is calculated for each model.

| Model | Random structure | AIC | χ² | d.f. | *P* | Dispersion parameter |
| --- | --- | --- | --- | --- | --- | --- |
| **1** | **no random term** | **6882.6** |  |  |  | 1.201 |
| 2 | 1\|individual | 6616.7 | 265.9 | 1 | < 0.0001 | 0.990 |
| 3 | 1\|replicate | 6785.7 | 97.27 | 1 | < 0.0001 | 1.192 |
| 4 | 1\|individual + 1\|replicate | 6605.2 | 278.8 | 2 | < 0.0001 | 0.986 |
|  |  |  |  |  |  |  |
| **4** | **1\|individual + 1\|replicate** | **6605.7** |  |  |  | 0.986 |
| 2 | 1\|individual | 6616.7 | 12.94 | 1 | 0.0003 | 0.990 |
| 3 | 1\|replicate | 6785.7 | 181.6 | 1 | < 0.001 | 1.192 |

**Table S3.** Model selection based on the Deviance Information Criterion (DIC) (MCMCglmm R package; Hadfield 2010). A higher DIC when the term is deleted from the global model indicates rejection of the simpler model in favor of the more complicated model.

| Deleted terms | DIC | ΔDIC |
| --- | --- | --- |
| None | 6249.763 | 117.127 |
| Local density * Neighboring density | 6277.112 | 27.349 |
| Local density * Local sex ratio | 6268.819 | 19.056 |
| Neighboring density * Local sex ratio | 6263.749 | 13.986 |
| Neighboring density * Neighboring sex ratio | 6260.376 | 10.613 |
| Sex (Male) * Local sex ratio | 6291.261 | 41.498 |
| Times | 6556.726 | 306.963 |
| Local density | 6363.976 | 114.213 |
| Neighboring density | 6361.733 | 111.97 |
| Sex | 6368.915 | 119.152 |
| Local sex ratio | 6382.850 | 133.087 |
| Neighboring sex ratio | 6496.515 | 246.752 |
| Times² | 6399.315 | 149.552 |

**S4 Table.** **Estimates of the best GLMMs (backward selection using likelihood ratio tests) across the range of *Δt* values from 5 to 20 min.** With the exception of a *Δt* value of 5 min, results were consistent across the range of *Δt* values that were used to produce the data describing real-time dynamics and the best GLMMs were qualitatively similar although there were some discrepancies in the absolute values of the estimated parameters (see Table S5). The “-“ symbol indicates that the variable was not in the best GLMM.

| **ΔT (minutes)** | **5** | | | | **10** | | | | |
| --- | --- | --- | --- | --- | --- | --- | --- | --- | --- |
| **Fixed effects** | **Estimate** | **sd** | **Z value** | **Pr(>\|z\|)** | **Estimate** | **sd** | | **Z value** | **Pr(>\|z\|)** |
| Intercept | -4.3899000 | 0.2948000 | -14.89 | < 0.0001 | -3.5416000 | 0.2891000 | | -12.25 | < 0.0001 |
| Times | -0.0000210 | 0.0000014 | -15.40 | < 0.0001 | -0.0000206 | 0.0000014 | | -15.09 | < 0.0001 |
| Local density | -0.0002976 | 0.0000977 | -3.05 | 0.00231 | -0.0003550 | 0.0000990 | | -3.58 | 0.000335 |
| Neighboring density | 0.0011613 | 0.0001209 | 9.61 | < 0.0001 | 0.0011550 | 0.0001474 | | 7.83 | < 0.0001 |
| Sex (Male) | -2.2495000 | 0.2335000 | -9.63 | < 0.0001 | -1.7779000 | 0.2291000 | | -7.76 | < 0.0001 |
| Local sex ratio | 0.4926000 | 0.4219000 | 1.17 | 0.24301 | 0.7700000 | 0.4210000 | | 1.82 | 0.067376 |
| Neighboring sex ratio | 2.6892000 | 0.2326000 | 11.56 | < 0.0001 | 2.3209000 | 0.2328000 | | 9.968 | < 0.0001 |
| Times² | 0.0000000 | 0.0000000 | 7.83 | < 0.0001 | 0.0000000 | 0.0000000 | | 7.47 | < 0.0001 |
| Local density * Neighboring density | 0.0000004 | 0.0000001 | 5.75 | < 0.0001 | 0.0000004 | 0.0000001 | | 5.85 | < 0.0001 |
| Local density * Local sex ratio | -0.0007424 | 0.0001567 | -4.74 | < 0.0001 | -0.0007128 | 0.0001639 | | -4.34 | < 0.0001 |
| Neighboring density * Local sex ratio | -0.0012504 | 0.0001761 | -7.10 | < 0.0001 | -0.0010518 | 0.0001822 | | -5.77 | < 0.0001 |
| Neighboring density * Neighboring sex ratio | - | - | - | - | -0.0003624 | 0.0001107 | | -3.27 | 0.001058 |
| Sex (Male) * Local sex ratio | 4.3106000 | 0.4105000 | 10.50 | < 0.0001 | 3.318000 | 0.400300 | | 8.29 | < 0.0001 |
| **ΔT (minutes)** | **15** | | | | **20** | | | | |
| **Fixed effects** | **Estimate** | **sd** | **Z value** | **Pr(>\|z\|)** | **Estimate** | | **sd** | **Z value** | **Pr(>\|z\|)** |
| Intercept | -3.5427 | 0.3345 | -10.59 | < 0.0001 | -3.4934 | | 0.3547 | -9.84 | < 0.0001 |
| Times | -0.01225266 | 0.000848 | -14.45 | < 0.0001 | -0.012366 | | 0.000857 | -14.43 | < 0.0001 |
| Local density | -0.028021 | 0.010676 | -2.62 | 0.008671 | -0.024412 | | 0.011316 | -2.15 | 0.030984 |
| Neighboring density | 0.072378 | 0.018968 | 3.81 | 0.000136 | 0.071716 | | 0.020016 | 3.58 | 0.00034 |
| Sex (Male) | -1.6464 | 0.2366 | -6.95 | < 0.0001 | -1.5658 | | 0.2437 | -6.42 | < 0.0001 |
| Local sex ratio | 1.2076 | 0.4637 | 2.60 | 0.009199 | 1.6397 | | 0.4845 | 3.38 | 0.000713 |
| Neighboring sex ratio | 2.4472 | 0.295 | 8.29 | < 0.0001 | 2.5074 | | 0.311 | 8.06 | < 0.0001 |
| Times² | 0.0000085 | 0.0000012 | 7.03 | < 0.0001 | 0.0000087 | | 0.0000012 | 7.02 | < 0.0001 |
| Local density * Neighboring density | 0.00417363 | 0.000749 | 5.57 | < 0.0001 | 0.0041664 | | 0.00077 | 5.40 | < 0.0001 |
| Local density * Local sex ratio | -0.081823 | 0.017627 | -4.64 | < 0.0001 | -0.089406 | | 0.018255 | -4.89 | < 0.0001 |
| Neighboring density * Local sex ratio | -0.100994 | 0.020072 | -5.03 | < 0.0001 | -0.105005 | | 0.021101 | -4.97 | < 0.0001 |
| Neighboring density * Neighboring sex ratio | 0.056783 | 0.020724 | 2.74 | 0.006146 | 0.061391 | | 0.021809 | 2.81 | 0.004878 |
| Sex (Male) * Local sex ratio | 3.0802 | 0.4174 | 7.37 | < 0.0001 | 2.8678 | | 0.4296 | 6.67 | < 0.0001 |

**S5 Table.** **Estimates of the best GLMMs (backward selection using likelihood ratio tests) across the range of *Δt* values from 25 to 60 min.** With the exception of a *Δt* value of 5 min, results were consistent across the range of *Δt* values that were used to produce the data describing real-time dynamics and the best GLMMs were qualitatively similar although there were some discrepancies in the absolute values of the estimated parameters (see Table S4).

| **ΔT (minutes)** | **25** | | | | **30** | | | | |
| --- | --- | --- | --- | --- | --- | --- | --- | --- | --- |
| **Fixed effects** | **Estimate** | **sd** | **Z value** | **Pr(>\|z\|)** | | **Estimate** | **sd** | **Z value** | **Pr(>\|z\|)** |
| Intercept | -3.1857000 | 0.3601 | -8.84 | < 0.0001 | | -2.8875 | 0.3614 | -7.99 | < 0.0001 |
| Times | -0.01215733 | 0.000886 | -13.72 | < 0.0001 | | -0.01193 | 0.000868 | -13.73 | < 0.0001 |
| Local density | -0.020231 | 0.01133 | -1.78 | 0.07416 | | -0.027134 | 0.011582 | -2.34 | 0.01914 |
| Neighboring density | 0.047711 | 0.020894 | 2.28 | 0.02240 | | 0.040481 | 0.021354 | 1.89 | 0.05799 |
| Sex (Male) | -1.5206 | 0.2476 | -6.14 | < 0.0001 | | -1.356 | 0.2492 | -5.44 | < 0.0001 |
| Local sex ratio | 1.6498 | 0.4989 | 3.30 | 0.00094 | | 1.4648 | 0.5002 | 2.92 | 0.00341 |
| Neighboring sex ratio | 2.2325 | 0.3122 | 7.15 | < 0.0001 | | 2.1741 | 0.3217 | 6.75 | < 0.0001 |
| Times² | 0.0000084 | 0.0000013 | 6.43 | < 0.0001 | | 0.0000079 | 0.0000013 | 6.28 | < 0.0001 |
| Local density * Neighboring density | 0.00470673 | 0.000795 | 5.92 | < 0.0001 | | 0.0049605 | 0.000812 | 6.10 | < 0.0001 |
| Local density * Local sex ratio | -0.103044 | 0.018738 | -5.49 | < 0.0001 | | -0.091559 | 0.019458 | -4.70 | < 0.0001 |
| Neighboring density * Local sex ratio | -0.094151 | 0.021732 | -4.33 | < 0.0001 | | -0.079584 | 0.022266 | -3.57 | 0.000351 |
| Neighboring density * Neighboring sex ratio | 0.091441 | 0.023867 | 3.83 | 0.00012 | | 0.085112 | 0.024094 | 3.53 | 0.000412 |
| Sex (Male) * Local sex ratio | 2.774 | 0.4368 | 6.35 | < 0.0001 | | 2.4389 | 0.4414 | 5.52 | < 0.0001 |
| **ΔT (minutes)** | **45** | | | | | **60** | | | |
| **Fixed effects** | **Estimate** | **sd** | **Z value** | **Pr(>\|z\|)** | | **Estimate** | **sd** | **Z value** | **Pr(>\|z\|)** |
| Intercept | -2.71602 | 0.3978 | -6.82 | < 0.0001 | | -2.2514 | 0.4293 | -9.848 | < 0.0001 |
| Times | -0.01138785 | 0.000877 | -12.98 | < 0.0001 | | -0.011082 | 0.000883 | -14.432 | < 0.0001 |
| Local density | -0.0009291 | 0.013628 | -0.06 | 0.94564 | | -0.007297 | 0.016112 | -2.157 | 0.03098 |
| Neighboring density | 0.0343447 | 0.023429 | 1.46 | 0.14267 | | 0.005834 | 0.025328 | 3.583 | 0.0003 |
| Sex (Male) | -1.3172 | 0.269 | -4.89 | < 0.0001 | | -1.4493 | 0.2911 | -6.425 | < 0.0001 |
| Local sex ratio | 1.96619 | 0.5561 | 3.53 | 0.0004 | | 1.6733 | 0.596 | 3.385 | 0.000713 |
| Neighboring sex ratio | 1.90558 | 0.3472 | 5.48 | < 0.0001 | | 1.8626 | 0.3866 | 8.063 | < 0.0001 |
| Times² | 0.0000071 | 0.0000013 | 5.44 | < 0.0001 | | 0.0000065 | 0.0000014 | 7.02 | < 0.0001 |
| Local density * Neighboring density | 0.005068284 | 0.000892 | 5.68 | < 0.0001 | | 0.0064855 | 0.000978 | 5.408 | < 0.0001 |
| Local density * Local sex ratio | -0.142167 | 0.022927 | -6.2 | < 0.0001 | | -0.149684 | 0.027893 | -4.898 | < 0.0001 |
| Neighboring density * Local sex ratio | -0.0855717 | 0.024298 | -3.52 | 0.00042 | | -0.066041 | 0.025799 | -4.976 | < 0.0001 |
| Neighboring density * Neighboring sex ratio | 0.1001716 | 0.027199 | 3.68 | 0.00023 | | 0.114134 | 0.029553 | 2.815 | 0.004878 |
| Sex (Male) * Local sex ratio | 2.30239 | 0.4778 | 4.81 | < 0.0001 | | 2.5195 | 0.5197 | 6.676 | < 0.0001 |
