# Supplementary material for: Local and neighboring patch conditions alter sex‐specific movement in banana weevils
Source: Ecol Evol. 2015 Nov 20;5(23):5735–43. doi: 10.1002/ece3.1818 (PMC4813121; doi:10.1002/ece3.1818)
Supplement: Supplementary file 2 — Figure S1. Experimental design for the study of the behavior of Cosmopolites sordidus, the banana weevil. Figure S2. Influence of the local densities and local sex ratio (number of males/number of individuals) on the probability of Cosmopolites sordidus movement. Figure S3. Influence of the neighboring densities and local sex ratio (number of males/number of individuals) on the probability of Cosmopolites sordidus movement. Figure S4. Influence of the neighboring densities and neighboring sex ratio (number of males/number of individuals) on the probability of Cosmopolites sordidus movement. [file ECE3-5-5735-s002.docx]

**
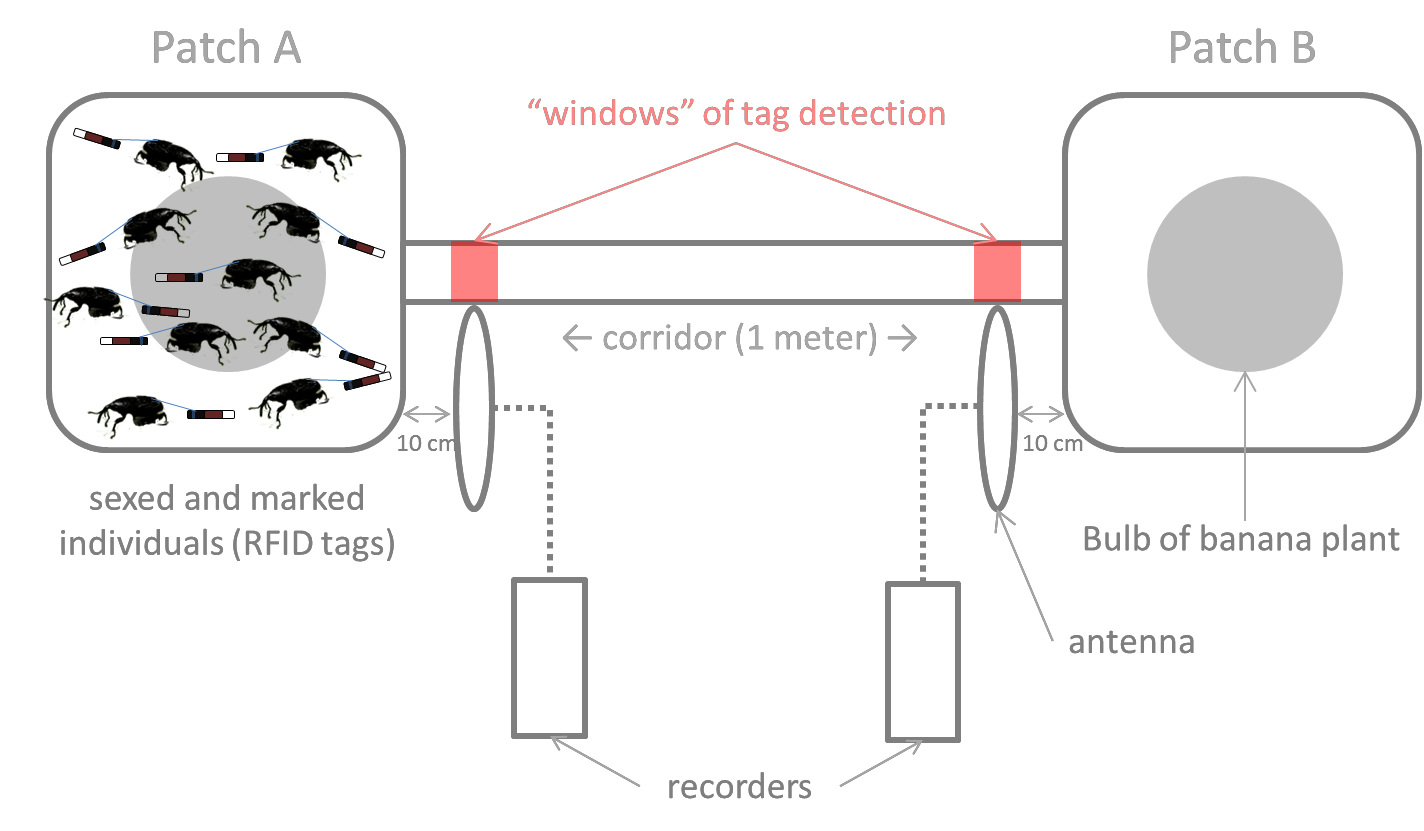
 S1 Fig. Experimental design for the study of the behavior of *Cosmopolites sordidus*, the banana weevil.**

**
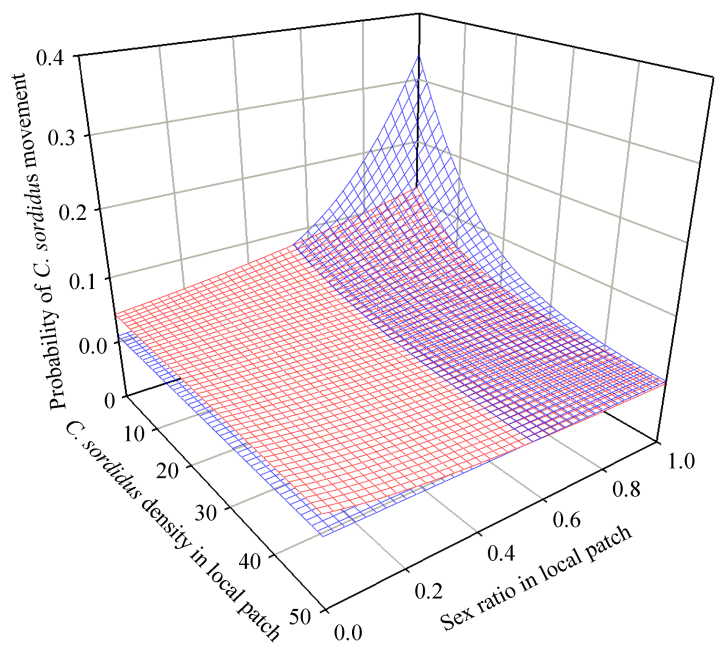
**

**Figure S2. Influence of the local densities and local sex ratio (number of males/number of individuals) on the probability of *C. sordidus* movement.** Red surface: movement response of females. Blue surface: movement response of males. The time was set at 120 min after the start of darkness, the neighboring density was set at 20 individuals, and the sex ratio (number of males/number of weevils) in the neighboring patch was set at 0.5.


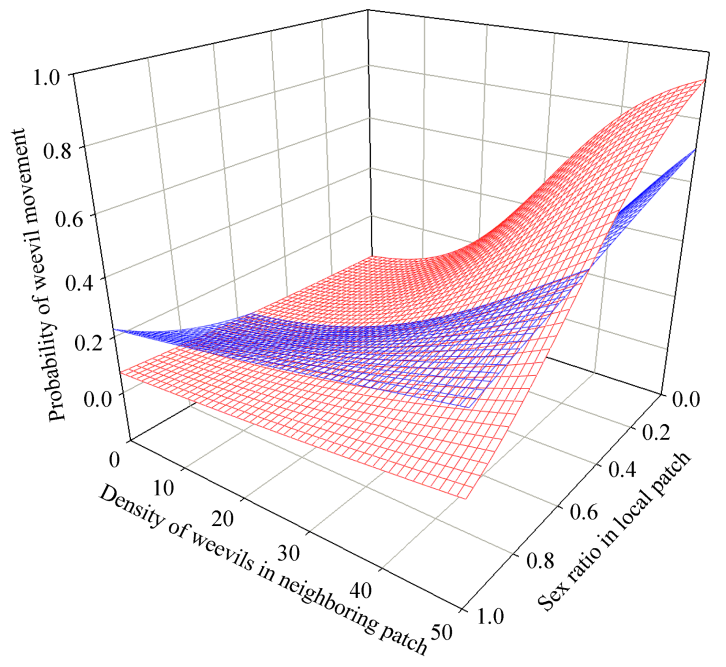


**Figure S3. Influence of the neighboring densities and local sex ratio (number of males/number of individuals) on the probability of *C. sordidus* movement.** Red surface: movement response of females. Blue surface: movement response of males. The time was set at 120 min after the start of darkness, the local density was set at 20 individuals, and the sex ratio (number of males/number of weevils) in the neighboring patch was set at 0.5.


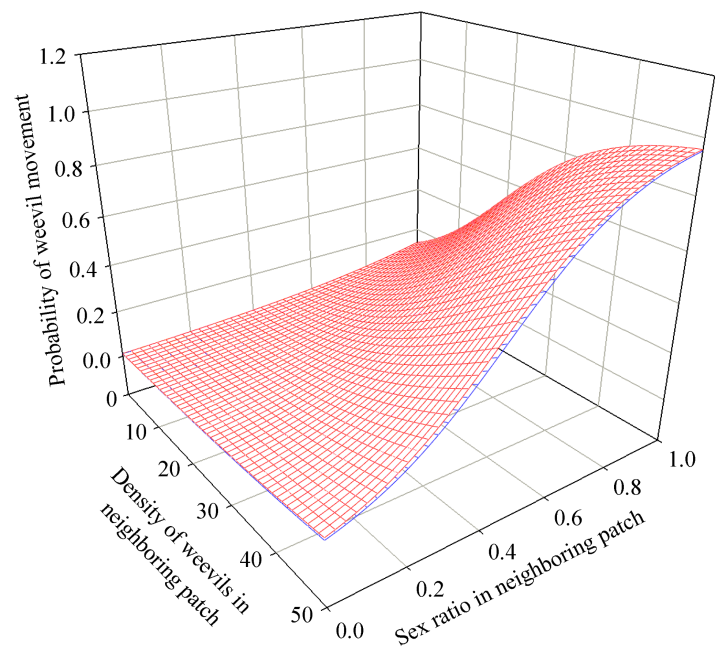


**Figure S4. Influence of the neighboring densities and neighboring sex ratio (number of males/number of individuals) on the probability of *C. sordidus* movement.** Red surface: movement response of females. Blue surface: movement response of males. The time was set at 120 min after the start of darkness, the local density was set at 20 individuals, and the sex ratio (number of males/number of weevils) in the local patch was set at 0.5.
